# Forest fire monitoring *via* uncrewed aerial vehicle image processing based on a modified machine learning algorithm

**DOI:** 10.3389/fpls.2022.954757

**Published:** 2022-10-17

**Authors:** Shaoxiong Zheng, Peng Gao, Xiangjun Zou, Weixing Wang

**Affiliations:** ^1^College of Electronic Engineering, South China Agricultural University, Guangzhou, China; ^2^Guangdong Engineering Research Center for Monitoring Agricultural Information, Guangzhou, China; ^3^Guangdong Laboratory for Lingnan Modern Agriculture, College of Engineering, South China Agricultural University, Guangzhou, China; ^4^Foshan-Zhongke Innovation Research Institute of Intelligent Agriculture and Robotics, Foshan, China

**Keywords:** forest fire insurance, BP neural network, image recognition, image segmentation, flame pixel

## Abstract

Forests are indispensable links in the ecological chain and important ecosystems in nature. The destruction of forests seriously influences the ecological environment of the Earth. Forest protection plays an important role in human sustainable development, and the most important aspect of forest protection is preventing forest fires. Fire affects the structure and dynamics of forests and also climate and geochemical cycles. Using various technologies to monitor the occurrence of forest fires, quickly finding the source of forest fires, and conducting early intervention are of great significance to reducing the damage caused by forest fires. An improved forest fire risk identification algorithm is established based on a deep learning algorithm to accurately identify forest fire risk in a complex natural environment. First, image enhancement and morphological preprocessing are performed on a forest fire risk image. Second, the suspected forest fire area is segmented. The color segmentation results are compared using the HAF and MCC methods, and the suspected forest fire area features are extracted. Finally, the forest fire risk image recognition processing is conducted. A forest fire risk dataset is constructed to compare different classification methods to predict the occurrence of forest fire risk to improve the backpropagation (BP) neural network forest fire identification algorithm. An improved machine learning algorithm is used to evaluate the classification accuracy. The results reveal that the algorithm changes the learning rate between 0.1 and 0.8, consistent with the cross-index verification of the 10x sampling algorithm. In the combined improved BP neural network and support vector machine (SVM) classifier, forest fire risk is recognized based on feature extraction and the BP network. In total, 1,450 images are used as the training set. The experimental results reveal that in image preprocessing, image enhancement technology using the frequency and spatial domain methods can enhance the useful information of the image and improve its clarity. In the image segmentation stage, MCC is used to evaluate the segmentationresults. The accuracy of this algorithm is high compared with other algorithms, up to 92.73%. Therefore, the improved forest fire risk identification algorithm can accurately identify forest fire risk in the natural environment and contribute to forest protection.

## Introduction

Forests are vital links in the ecological chain and essential ecosystems in nature. Damaged forests seriously affect the ecological environment of the Earth (Han et al., 2018). Protecting the forest environment plants plays a critical role in sustainable human development, and the most important aspect of protecting forest plants is preventing forest fires. If a forest fire is not monitored and warned of in time, it burns down trees, reduces forest accumulation, and causes soil erosion and vegetation damage (Ryan et al., 2021). Forest fires can also destroy understory plant resources and cause irreparable losses to rich wild plant resources. Developing diversified forest fire risk monitoring methods is necessary to reduce the adverse effects of forest fires (Cai et al., 2018).

Developed countries have conducted substantial research on monitoring forest fire disasters to reduce the losses caused by forest fires. For example, Canada has a vast territory with an average of thousands of forest fires daily, but no major fire has occurred in recent decades. Japan has also made some achievements in forest fire disaster monitoring, and there have been no major fires. These are inseparable from the rapid development of forest fire monitoring technology (Van Hoang et al., 2020). Currently, there are two main monitoring methods for forest fire risk at home and abroad. The first is through ground monitoring, including infrared, video, and radar monitoring, and the other is air monitoring, primarily through satellite, microwave, and infrared monitoring (Hu et al., 2022).

The recognition and monitoring of forest fire risk images using a machine learning algorithm have been successfully performed in recent years to realize the timely monitoring of forest fires. First, to extract the features of the forest fire risk image, we segment the image and then classify it according to the features.

Thach et al. (2018) analyzed the spatial pattern of tropical forest fire risk using random forest and the multilayer perceptron neural network and used the Pearson correlation method to evaluate the correlation between the variables and forest fire. In total, three forest fire risk models, the support vector machine (SVM) classifier, random forest, and multilayer perceptron neural network, were trained and verified (Thach et al., 2018).

Lim et al. (2019) constructed two forest fire risk prediction models based on satellite fire data and medium-resolution imaging spectrometry data monitored by the Korean Forestry Administration. They analyzed the spatial autocorrelation between the fire frequency and intensity of the two data types using a semivariogram. The accuracy and performance of the model are good (Lim et al., 2019).

Balling improved the processing and analyzability of the forest image returned by the uncrewed aerial vehicle (UAV) by designing the UAV forest fire prevention system, forest fire image monitoring algorithm, and intelligent landing gear system. This method realized the real-time monitoring of forest fire and improved the digitization and automation of forest fire early warning and prevention (Balling et al., 2021).

Devotta et al. (2021) proposed an improved recognition and positioning algorithm based on the color index. Combined with the forest fire monitoring recognition algorithm of the UAV, it can process the video image data returned during the flight of the aircraft in real time, which can monitor and recognize forest fire risk and accurately judge its location (Devotta et al., 2021).

However, image processing technology based on machine learning can extract and analyze the image features of forest fire risk and effectively identify the risk of a forest fire. Zhang et al. (2019) built a forest fire prediction model based on the convolutional neural network structure suitable for forest fire sensitivity prediction.

Moayedi et al. (2020) adopted a hybrid evolutionary algorithm to realize the approximate and reasonable task of this forest fire environmental threat. A total of three fuzzy meta-heuristic algorithms, the genetic algorithm, particle swarm optimization algorithm, and differential evolution algorithm, were used to construct a sensitive area model of the forest fire. The results reveal that the optimized structure can replace the traditional forest fire prediction model (Moayedi et al., 2020).

Ghali et al. (2021) used deep learning technology to establish a deep learning convolutional transfer learning feature extraction network. Ghali also explored the correlation of the popular allocation standard of subspace learning and designed the deep convolution and domain adaptive sample classification algorithm. The experimental effect was good (Ghali et al., 2021).

The aforementioned methods obtain forest canopy image information using UAVs or video surveillance; analyze the complex characteristics of smoke, flame, and other images by processing forest fire risk images; and build a forest fire risk monitoring and early warning model. The model can predict the time of fire risk, direction of fire spread, and fire intensity. However, few researchers have studied high-precision and lightweight backpropagation neural network (BPNN) models. The BPNN and SVM algorithm are combined to build the forest fire risk identification algorithm MD-BPNN based on an improved BPNN to improve the efficiency of forest fire risk identification.

The organizational structure of this article is as follows: In section “Improvement of forest fire identification algorithm,” we recognize forest fire risk images. In section “Forest fire risk image recognition,” we propose a color segmentation model of forest fire insurance. Section “Color segmentation model of forest fire insurance” introduces the improvement of a BP neural network forest fire identification algorithm and makes an experimental analysis. Finally, section “Conclusion” summarizes the conclusions.

## Improvement of forest fire identification algorithm

### Steps of the algorithm

Michael et al. (2021) proposed that the BPNN primarily comprises forward signal propagation and reverse error signal propagation. During the forward propagation of the signal, the difference between the output signal and expected output value is calculated. The error signal is transmitted in reverse through the output when a large error occurs, and the value of each layer is modified to make the actual output close to the expected output.

**FIGURE 1:**
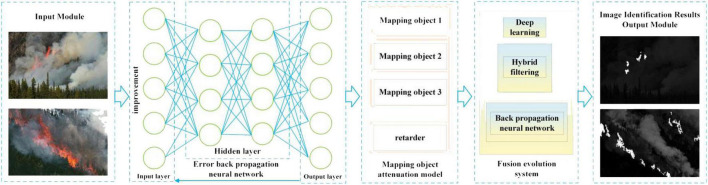
Modified machine learning algorithm network model structure.

In this study, the image processing steps through the layers are illustrated in Figure 1, and the output of the image recognition result is provided. When the input layer has a reverse propagation method, it may affect the input and output of other layers (Al-Zebda et al., 2021). The image enters the input, hidden, and output layers and is processed by the BPNN. Finally, the processed image is generated in the output layer. The MapReduce fusion deep learning neural network based on BP comprises the input, hidden, and output layers, as depicted in Figure 1. It is trained in two ways: the forward propagation deep learning method and BP deep learning process. The input of the former method affects the other layers. The possible errors in the input data of the output layer are corrected through BP.

According to the weight vector space, gradient descent technology enhances the search technology and reduces the error rate. In the hidden layer, *N* is the number of neurons. The hidden layer can be regarded as the input of the output layer, and whether the result is correct can be observed in its evaluation. The series *N* = [*1*,*2*,*3*,⋯⋯,*10*] indicates the largest prediction result found. The series [*x_1_,x_2_*,⋯⋯,*x_n_*] represents input variables. The series [*w_1_,w_2_*,⋯⋯*w_n_*] denotes the weight between the hidden and input layers. Finally, the series [*v_1_,v_2_*,⋯⋯,*v_n_*] is the weight assigned to hidden and output layers. In addition, *_Y(t)_* is a predictable output, and the transfer function is used to describe the nonlinear problem, expressed as follows:


(1) $$y=f\,\left(x\right)=\frac{1}{1+e^{-x}}$$

The hidden layer z is the output expressed as follows:


(2) $$z=f_{1}\,\left(\sum w_{i}\,x_{i}\right),$$

where_*w_1_,w_2_,w_3_……w_n_*_ represent the weights of the hidden and input layers:


(3) $$u=f_{2}\,\left(\sum u_{k}\,z_{k}\right).$$

After the forward propagation process is completed, the error signal e is formed by the tasks of *u* and *y*(t) for the purpose of


(4) $$u=1/2\,{\left(\sum y_{i}\,{\left(t\right)}_{u}-z_{i}\right)}^{2}$$

### Modified machine learning algorithm based on backpropagation neural network

In this study, the improvement steps of the deep learning BP neural network forest fire identification algorithm are as follows (Kukuk and Kilimci, 2021).

Step 1: Set algorithm parameters, including the initial weight diversity feature (*W*), that is, the weight between [−*w*, *w*] ranges. The lowest initial range here is [−1, 1].

Number of hidden layers: The number is set to 1 in this study.

Number of nodes in the hidden layer: The number of nodes in the hidden layer is less than the number of training samples.

Number of training cycles: One training cycle can improve the accuracy of description by scanning the records of the training set. At the cost of time, the accuracy may be reduced, but the use time will be reduced.

Error tolerance: It specifies that the error tolerance in the restatement is low. In terms of characteristics, error tolerance is a small-cost event with a diversity from 0 to 1.

Hidden layer sigmoid: It generates each hidden node through a sigmoid function. It can transform the continuous real value of the input into the output between 0 and 1.

Serious error: It avoid serious errors in network training (Mohammed, 2022).

Step 2: The output of the active function marked in the render parameters for each layer is calculated.

Activation function: It is responsible for mapping the input of neurons to the output.

Learning rate: The logarithm is kept in the range of [0.5∼0.8] at the beginning of discovery.

Number of training cycles: This is the only scan of all images in the training set.

Step 3: Once an error is found in the output layer, the error between all the obtained outputs and the selected outputs is calculated.

Step 4: The weight extension error gradient is adjusted on each epoch.

Step 5: The deep training mode is used to obtain the output. The depth training mode is expanded from the depth training set, allowing the functions marked in the aforementioned parameter list to be activated.

Step 6: Depth fusion is performed in the estimation function.

Step 7: The incorrect information of deep fusion is ignored, and the model is trained.

**FIGURE 2:**
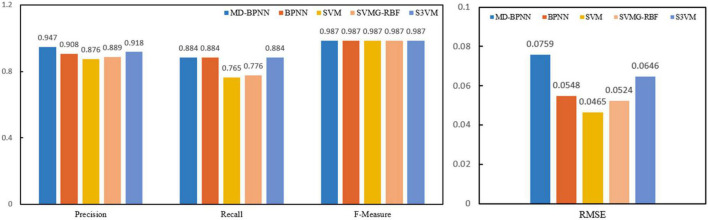
Performance comparison of all network models without sampling.

**FIGURE 3:**
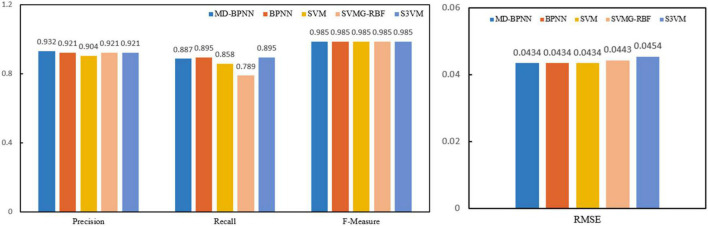
Performance comparison of all network models with resampling.

Experimental results: In the cluster (about five nodes), an 8.00 GB i3 CPU and 2.8 GHz of RAM are used. A forest fire risk dataset was constructed to compare different methods to predict forest fire risk. The forest fire risk monitoring dataset includes forest fire risk images collected by UAVs on the ground and forest fire risk images searched on the internet. Figure 2 presents the calculation results of the non-sampling fusion-level depth learning using several algorithms, and Figure 3 depicts the calculation results of the fusion-level depth learning perceptron with resampling.

Figure 2 compares the algorithm performance using the network model without resampling. The recognition accuracy of the MD-BPNN algorithm is 0.947, which is the highest compared with the accuracy of other algorithms. The recall rate of the MD-BPNN algorithm is 0.884, and the F-measure is 0.984. The root mean square error (RMSE) is 0.0759. According to Figure 3, the algorithm performance is compared using the network model with resampling. The recognition accuracy of the MD-BPNN algorithm is 0.932, and the recall rate is 0.887. Furthermore, the F-measure is 0.985, and the RMSE is 0.0434.

The data in the table reveal the consistency measurement of the proposed algorithm. The algorithm changes the learning rate between 0.1 and 0.8, consistent with the cross-index verification of the 10x sampling algorithm.

### Improved machine learning algorithm and support vector machine classifier

**FIGURE 4:**
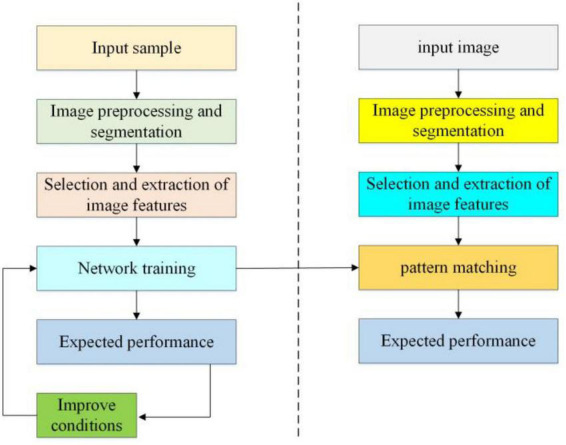
Image recognition steps based on backpropagation neural network.

This article presents an improved machine learning algorithm and SVM classifier to recognize forest fire risk images. This method takes the color and shape of the fire image as the criterion and combines a variety of features. This method first extracts the color features of the flame according to the composition of the flame color to reduce some significant interference (Tang et al., 2022). Second, by constructing the multi-dimensional vector of color and shape, the shape characteristic parameters of the target area are calculated. The image recognition steps based on the BPNN are shown in Figure 4.

The input layer of the forest fire risk recognition neural network based on the BPNN is composed of extracted features, including color moments, roundness ρ of the suspicious area, and the angle *N* of flame.

According to the different backgrounds of forest fire risk images, the distribution of *y* is *y* = [*1*, *0*]. *y* = [*0*, *1*, *0*], and*y* = [*0*, *1*]. According to Equation (5), the node in the hidden layer is 8.


(5) $$y=\frac{\frac{1}{2}\,c\times r^{2}+\frac{3}{2}\,c\times r-1}{c+r}$$

After the number of nodes in the network hidden layer is determined, the forest fire risk image recognition steps are as follows:

Initialization: The input vector is *X* = [*x_1_*, ⋯, *x_n_*], the output vector is *Y* = [*y_1_*, ⋯, *y_k_*], and the hidden neuron vector is *M* = [*m_1_*, ⋯, *m_j_*].

Forward propagation: According to $S_{j}=\sum_{1}^{i}X_{i}\,W_{\mathrm{ij}}$, the fitness of hidden layer is $f\,\left(x\right)=\tanh\,\left(x\right)=\frac{e^{x}-e^{-x}}{e^{x}+e^{-x}}$, so the output function of the hidden layer is *M*_*j*_ = *f* (*S _i_*).

According to $Q_{k}=\sum_{1}^{j}M_{j}\,V_{\mathrm{jk}}$, the fitness of the output layer is $f\,\left(x\right)=\frac{1}{1+e^{-x}}$ and *Y*(*k*) = *f* (*Q _k_*).

Backpropagation: The actual output is *Y*(*k*), the expected output is *O*_*k*_, the mean square error is $E_{n}=\frac{1}{2}\,\sum_{1}{\left[O\,\left(k\right)-Y\,\left(k\right)\right]}^{2}$, and the sum of the mean square error is *E* = ∑_*n*=*1*_ *E*_*n*_. The learning error *d*_*k*_ of the input layer and the learning error *d*_*j*_ of the hidden layer are calculated, and the value of the weight is adjusted until the error disappears and is less than the expected error.

A total of 3,845 forest fire risk images which are taken in Longshan Forest Farm of Shaoguan, Guangdong (23 ° 12′N, 113 ° 22′E), and Lingyun Mountain of Foshan city (22 ° 57′N, 112 ° 46′E), are used as datasets. According to the ratio of 7:3, the datasets are divided into training sets and test sets. The datasets contain the image information of trees, lakes, roads, etc. in the forest environment taken by UAVs from above. The image information is collected under the conditions of sufficient light and low illumination. Longshan Forest Farm is located in Lechang city, Shaoguan, Guangdong Province. The forest farm mainly grows ecological public welfare forests and experimental forests such as Chinese fir, high-fat masson pine, bald cedar, eucalyptus, and rosemary. Lingyun Mountain is located in Gaoming district, Foshan city. Its vegetation consists of mainly masson pine, which is flammable and difficult to extinguish in case of fire. The environment of coniferous forest creates favorable conditions for the generation of crown fire.

Overall, 1,440 images are selected as the training samples, and 288 images are selected as the test samples, and the image samples are collected under the same background. Table 1 shows the parameters of some training samples.

**TABLE 1:** Parameters of some training samples.

I_1_	I_2_	I_3_	ρ	N	Y/N
0.0239	0.7301	0.4718	0.4572	0.2351	Y
0.1338	0.7471	0.5461	0.3473	0.1445	Y
0.2534	0.3548	0.0459	0.3151	0.6882	Y
0.4283	0.1352	0.3269	0.5277	0.9427	Y
0.0461	0.2451	0.1897	0.4281	0.8324	N

Table 2 lists the processing results. The results demonstrate that the image is considered a fire image if the output value exceeds 0.8.

**TABLE 2:** Training sample.

I_1_	I_2_	I_3_	ρ	N	Y/N	Result
0.1361	0.7472	0.5471	0.3463	0.1464	Y	0.9429
0.4282	0.1353	0.3269	0.5279	0.9429	Y	0.9141
0.5261	0.1746	0.0452	0.3142	0.7483	Y	0.6758
0.6419	0.0250	0.1887	0.4282	0.8323	Y	0.9943
0.0247	0.4301	0.6718	0.4527	0.2373	N	0.7703

## Forest fire risk image recognition

In UAV image forest fire monitoring, image preprocessing technology is an essential link. Due to the influence of environmental factors such as illumination and image background, the image acquisition process will reduce the quality and clarity of the collected image, resulting in the inability to truly reflect the details of the image (Sayad et al., 2019; Cawson et al., 2020; Hossain et al., 2020). The purpose of applying image preprocessing technology is to weaken or eliminate useless image information so as to retain and enhance useful information.

In order to better meet the training requirements of the BP neural network model, the original fire image data are converted into small images of the same size (Gaur et al., 2021; Lin et al., 2021; Wang et al., 2021). Preprocessing of image data is divided into the following five steps:

The original forest image data are randomly cropped to 256 × 256 and the image is randomly rotate at −15°∼15°.

The cropped forest fire risk image with size 224 × 224 is transformed into a tensor of 0∼1.

After converting from –1 to 1, the tensor is normalized from 0 to 1.

### Image enhancement

In the process of forest fire risk image recognition, the image needs to be enhanced (Abedi Gheshlaghi et al., 2021). The processing algorithm steps are as follows: First, the images are classified and divided into non-overlapping parts.


(6) $$\left[\begin{array}{c} {\mathrm{Gx}}^{\prime }\\ {\mathrm{Gy}}^{\prime } \end{array}\right]=\left[\begin{array}{c} {\mathrm{Gx}}^{2}-{\mathrm{Gy}}^{2}\\ 2\,G\,x\,G\,y \end{array}\right]$$

The average gradient vector ${\left[\bar{{\mathrm{Gx}}^{\prime }},\bar{{\mathrm{Gy}}^{\prime }}\right]}^{T}$ is calculated by using the following equation:


(7) $$\left[\begin{array}{l} \bar{Gx^{\prime }}\\ \bar{Gy^{\prime }} \end{array}\right]=\left[\begin{array}{l} \frac{1}{w\times w}\sum_{i=1}^{w}\sum_{i=1}^{w}\left(G_{x}^{2}\left(i,j\right)-G_{y}^{2}\left(i,j\right)\right)\\ \frac{1}{w\times w}\sum_{i=1}^{w}\sum_{i=1}^{w}\left(2G_{x}\left(i,y\right)G_{y}\left(i,y\right)\right) \end{array}\right]$$

In Equation (7), the size of the image area is *w* × *w*:


(8) φ={12⁢tan-1⁢G′y¯G′x+π2⁢tan-1⁢G′y¯G′x<012⁢tan-1⁢G′y¯G′x-π2⁢tan-1⁢G′y¯G′x≥0}

The frequency of the flame determines the filtering effect (Babu et al., 2019; Sayad et al., 2019). If the frequency is not appropriate, the filtered image will be greatly deformed, resulting in the suppression of some flame structures, so the filtered image has a blank position. The direction window is determined according to the flame direction, and each pixel in the window is projected to the baseline. In order to calculate the flame frequency of the image, it is necessary to calculate the distance between the projected crest and trough. The algorithm steps are as follows (Mao et al., 2018):

We divided the image into non-overlapping subblocks of *w* × *w*. Next, we calculated the average value of each point along the *w* direction, denoted as M[ K ].

Combined with the characteristics of forest fire risk images, this study has used the method of enhancement filtering to enhance the image quality, and the most commonly used enhancement image algorithm is Gabor filtering (Wang et al., 2019). This algorithm regards the image in the local area as a group of parallel, frequency, linear, and fixed direction images. The Gabor window function is used to locate and filter the flame in the image so as to enhance the flame information. It can be expressed as follows:


(9) $$G\,\left(x,y\right)=\frac{1}{2\,\pi \,\pi_{x}\,\sigma_{y}}\,\exp\,\left[-\frac{1}{2}\,\left(\frac{x^{2}}{\sigma_{x}^{2}}+\frac{y^{2}}{\sigma_{y}^{2}}\right)\right]\,\exp\,\left(-2\,\pi \,\pi \,\mathrm{fix}\right)$$

As can be seen from Equation (9), combined with the directional characteristics of the image rotation filter, image information can be enhanced during filtering (Sun et al., 2021).


(10) $$R\,\left(x,y\right)=\frac{1}{2\,\pi \,\pi_{x}\,\sigma_{y}}\,\exp\,\left[-\frac{1}{2}\,\left(\frac{x^{2}}{\sigma_{x}^{2}}+\frac{y^{2}}{\sigma_{y}^{2}}\right)\right]\,\cos\,\left(2\,\pi \,\pi \,\mathrm{fx}\right)$$

(11) $$V\,\left(x,y\right)=\frac{1}{2\,\pi \,\pi_{x}\,\sigma_{y}}\,\exp\,\left[-\frac{1}{2}\,\left(\frac{x^{2}}{\sigma_{x}^{2}}+\frac{y^{2}}{\sigma_{y}^{2}}\right)\right]\,\sin\,\left(2\,\pi \,\pi \,\mathrm{fx}\right)$$

(12) $$R\,\left(x,y,f,\varphi \right)=\frac{1}{2\,\pi \,\pi_{x}\,\sigma_{y}}\,\exp\,\left[-\frac{1}{2}\,\left(\frac{x_{\varphi }^{2}}{\sigma_{x}^{2}}+\frac{y_{\varphi }^{2}}{\sigma_{y}^{2}}\right)\right]\,\cos\,\left(2\,\pi \,{\mathrm{fx}}_{\varphi }\right)$$

**FIGURE 5:**
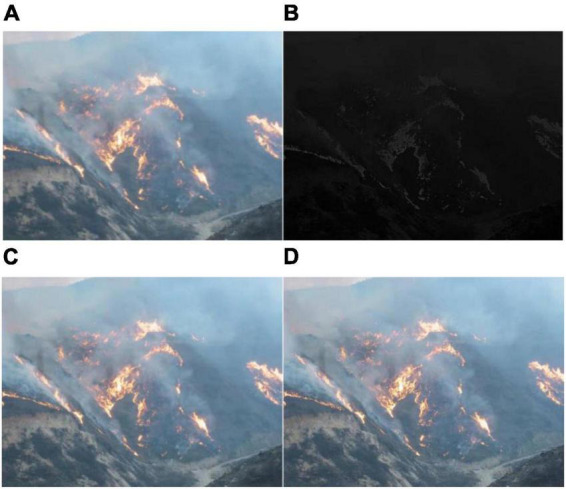
Effect picture after using various filtering processes: **(A)** Original drawing, **(B)** gabor filtering, **(C)** mean filtering, and **(D)** gaussian filtering.

In Equation (12), $\left[\begin{array}{l} x_{\varphi }\\ y_{\varphi } \end{array}\right]=\left[\begin{array}{l} cos\varphi \;sin\varphi \\ -sin\;cos\varphi \end{array}\right]\left[\begin{array}{l} x\\ y \end{array}\right]$, φ is the direction of the Gabor filter, [*x*_φ_, *y*_φ_] represents the angle of rotation along the axes x and y, and f is the frequency of the sine wave and plane wave (Hong et al., 2018). There are two methods to enhance image information: one is the frequency domain method, and the other is the spatial domain method, such as image gray transformation operation, image histogram correction operation, and image filtering operation (Sousa et al., 2020; Joardar et al., 2021). Figure 5 is the effect picture after Gabor filtering, mean filtering, and Gaussian filtering.

### Image morphological processing

Morphological processing of forest fire risk image data captured by UAVs can extract image components that are meaningful to the rendered area, realize further discrimination operation, and extract the edge features of the target object and the essential features of the connected area (Devotta et al., 2021).

**FIGURE 6:**
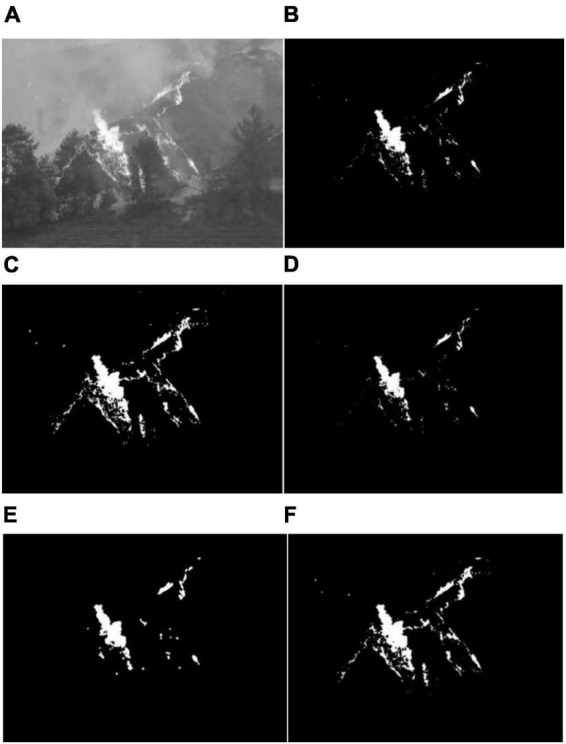
Image morphological processing results: **(A)** Original image, **(B)** binarization treatment, **(C)** expansion treatment, **(D)** corrosion treatment, **(E)** open operation processing, and **(F)** closed operation processing.

The basic operations of morphological image processing include expansion, corrosion, open operation, and close operation. In Figure 6A is the original (Figures 6B–F) are the comparison of the effects of several image morphological processing methods, such as binarization processing, expansion processing, corrosion processing, open operation, and closed operation (Wang et al., 2022).

Through the morphological processing of forest fire risk images, the image noise can be removed, the image shape can be simplified, the flame feature structure can be enhanced, and the flame information can be separated from the complex background.

## Color segmentation model of forest fire insurance

**FIGURE 7:**
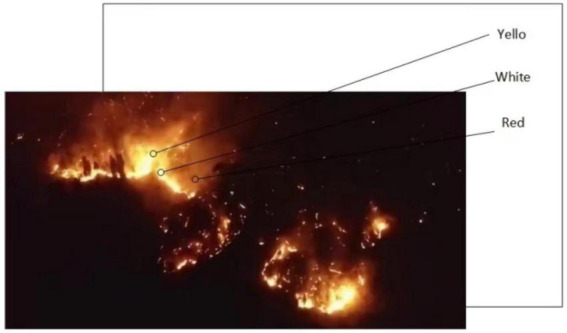
Flame color distribution.

In order to obtain a more detailed flame image, the flame pixels can be segmented in YCbCr color space and RGB color space, and the decision conditions can be obtained according to the two color spaces (Stankevich, 2020). In case of forest fire risk, the color of forest fire is quite different from the background color of the forest environment, and the characteristics are obvious. The main performance is that the color distribution of flame from outside to inside is red, yellow, and white. The color distribution of flame is shown in Figure 7.

### Pixel distribution characteristics of forest fire

**FIGURE 8:**
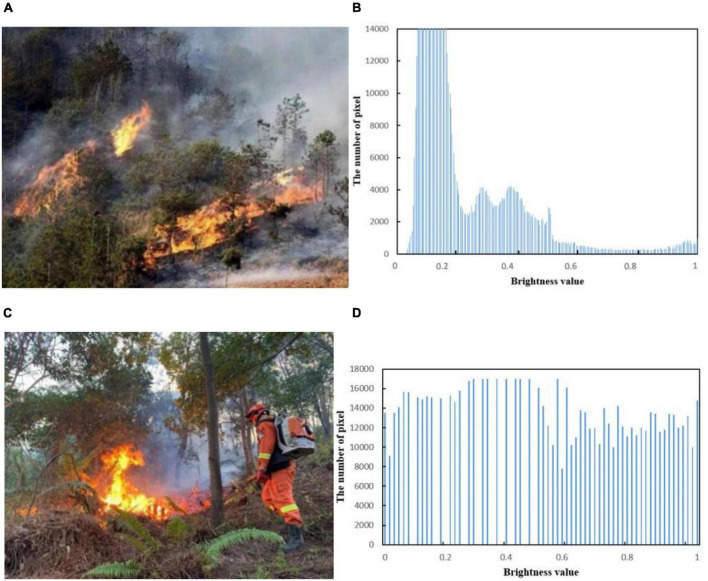
Flame brightness value: **(A)** Original image 1, **(B)** image 1 brightness value, **(C)** original image 2, and **(D)** image 2 brightness value.

In the actual forest fire risk monitoring process, when the shooting time is cloudy, the collected image information is dark. For general algorithms, it is usually difficult to achieve good results after direct processing. In order to improve the contrast effect of the image, the image needs to be preprocessed in advance. As shown in Figures 8A,B, the overall brightness value of the flame image is low. The color parameters of the HSV model include brightness, hue, and saturation (Van Le et al., 2021). The pixel value of the flame image is calculated. As shown in Figures 8C,D, the processed image has an obvious contrast effect and can meet the requirements of later image processing.

### Distribution of flame pixels

**FIGURE 9:**
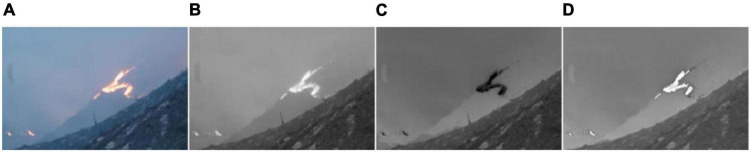
YCbCr color space separation image: **(A)** Original image, **(B)** Y channel separation, **(C)** Cb channel separation, and **(D)** Cr channel separation.

In Figures 9A–D are the flame image and the image separated by YCbCr channels.

**FIGURE 10:**
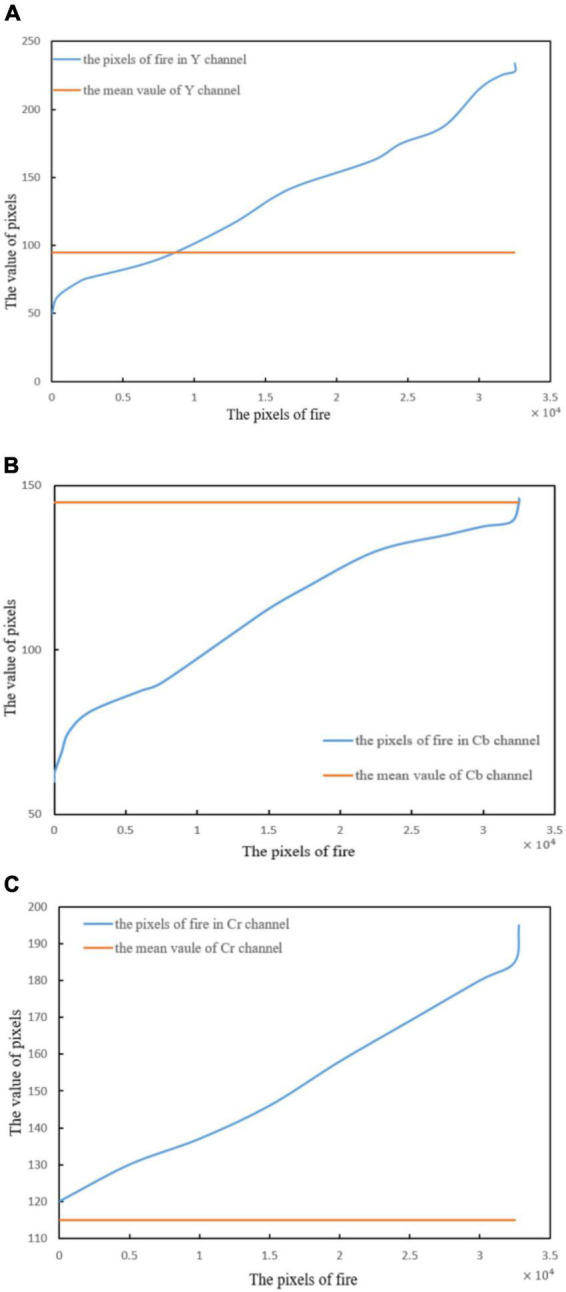
Pixel values of flames in different channels: **(A)** Flame pixel value of Y channel, **(B)** flame pixel value of Cb channel, and **(C)** flame pixel value of Cr channel.

This section compares the flame pixels of YCbCr channels with the average pixel value of the channel to describe the characteristics of flame pixels. In Figures 10A–C compare the flame pixel values in YCbCr channels with the average value of the corresponding channels (Mohammed, 2022).

As can be seen from Figure 10, in the three channels, the distribution rule of flame pixels can be expressed as follows (Sevinc et al., 2020; Kumar et al., 2021; Dharmawan et al., 2022):


(13) $$Y\,\left(i,j\right)>Y_{\mathrm{mean}},Y\,\left(i,j\right)>Y_{\mathrm{mean}}\,\mathrm{and}\,\mathrm{Cb}\,\left(i,j\right)<{\mathrm{Cb}}_{\mathrm{mean}}$$

where *Y_mean_Cb_mean_Cr_mean_* represent the average pixel value.

**FIGURE 11:**
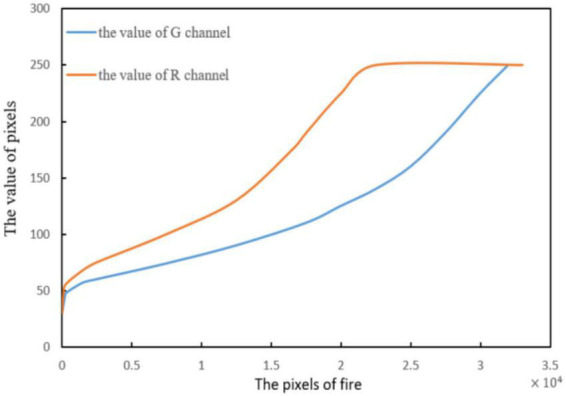
Value of pixels in the R and G channels.

As shown in Figure 11. The comparison results are as follows:


(14) R⁢(i,y)>G⁢(i,y)

### Color segmentation results and comparison

**FIGURE 12:**
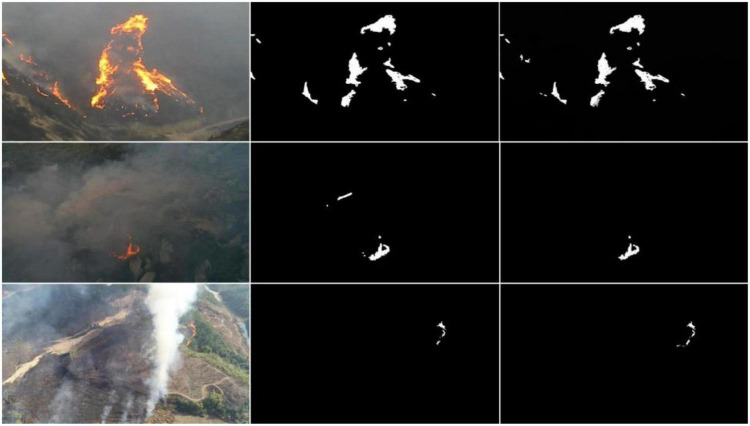
Segmentation flame image results.

This study uses the HAF (Equation 17) and MCC (Equation 18) methods to analyze the flame image information under low-light conditions and segment the flame color in the image. Figure 12 presents the results.

Figure 12 illustrates the segmentation results of the flame image. The left column is the original image, and the central column is the segmented flame image based on the general BPNN algorithm. The right column displays the results from the proposed MD-BPNN algorithm.

The segmentation quality of the region algorithm can be quantified through this index. We define the matching index calculation as follows:


(15) $$M=\sum j,\max_{i}\,\mathrm{Card}\,\left(R_{i}^{\mathrm{ref}}\,⋂R_{j}^{\mathrm{seg}}\right)\,\frac{\mathrm{Card}\,\left(R_{i}^{\mathrm{ref}}\,⋂R_{j}^{\mathrm{seg}}\right)}{\mathrm{Card}\,\left(R_{i}^{\mathrm{ref}}\,⋂R_{j}^{\mathrm{seg}}\right)}\times \rho_{j},$$

where *Card* represents the number of pixels, ρ_*j*_ denotes the weight, $R_{i}^{\mathrm{ref}}$ represents the *i*th manually segmented region, and $R_{j}^{\mathrm{seg}}$ denotes the *j*th region.

For the overlapping part, combined with the over-segmentation problem, the segmentation result region corresponds to the manual segmentation result region, and the following indexes are determined:


(16) $$\eta =\left\{ \begin{array}{c} \frac{{\mathrm{NR}}_{\mathrm{ref}}}{{\mathrm{NR}}_{\mathrm{seg}}},\mathrm{if}\,{\mathrm{NR}}_{\mathrm{seg}}\geq {\mathrm{NR}}_{\mathrm{ref}}\\ \log\,\left(1+\frac{{\mathrm{NR}}_{\mathrm{seg}}}{{\mathrm{NR}}_{\mathrm{ref}}}\right),\mathrm{otherwise} \end{array}\right.$$

The calculation equation of the final evaluation index HAF is as follows:


(17) $$\mathrm{HAF}=\frac{M+m\times \eta }{1+m}$$

**FIGURE 13:**
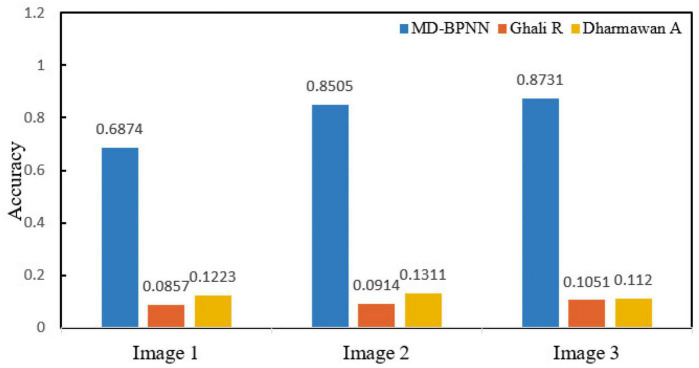
HAF evaluation index results.

In Equation (17), the weighting factor M plays an important role in judging the segmentation of the process, and its value is 0.5. In Figure 10, the HAF segmentation index comparison is shown in Figure 13.

From the test data, it can be seen that in the case of excessive segmentation and insufficient segmentation, the average accuracy rate of the MD-BPNN algorithm evaluated by using the HAF index is 80.37%, and the segmentation result still has good anti-interference performance. Compared with other algorithms, the performance is better, which enhances the applicability of the algorithm in multiple scenarios.

The Matthews coefficient is used to evaluate results, calculated as follows (27):


(18) $$\mathrm{MCC}={\frac{\left(\mathrm{TP}\times \mathrm{TN}\right)-\left(\mathrm{FP}\times \mathrm{FN}\right)}{\sqrt{\left(\mathrm{TN}+\mathrm{FN}\right)\,\left(\mathrm{TN}+\mathrm{FP}\right)\,\left(\mathrm{TP}+\mathrm{FN}\right)\,\left(\mathrm{TP}+\mathrm{FP}\right)}}}_{.}$$

**FIGURE 14:**
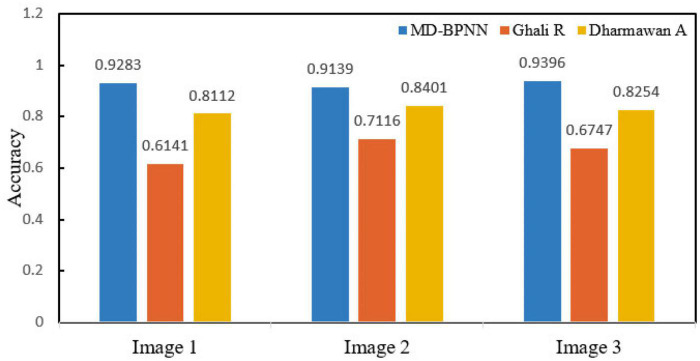
MCC evaluation index results.

In Equation (18), TP indicates a true positive, TN denotes a true negative, FP represents a false positive, and FN indicates a false negative. The MCC evaluation index results are presented in Figure 14.

By calculating the accuracy of the algorithm, the average accuracy of the MD-BPNN is 92.73%, which is better than other authors’ algorithms.

## Conclusion

According to the actual forest image recognition situation, this study improves the combination algorithm of the BP neural network algorithm and SVM classifier, models the recognition network, enhances the image and morphological processing through the training and learning of the recognition network, segments and extracts the features of the suspected forest fire area, improves the efficiency of forest fire recognition, and improves the stability of the network. The main contents are as follows:

(1) A forest fire risk dataset was constructed to compare different classification methods to predict forest fire risk. The results reveal that the algorithm changes the learning rate between 0.1 and 0.8, consistent with the cross-index verification of the 10x sampling algorithm.
(2) In the combination of an improved BP neural network and SVM classifier, forest fire risk is recognized based on feature extraction and a backpropagation network. A total of 1,450 images are used as training samples. The experimental results show that the recognition effect of fire risk images is good.
(3) By analyzing the forest fire image, the flame pixel values of the images on R and G channels are analyzed. The value of the former is higher than that of the latter.
(4) For the flame image under low lighting conditions, HAF and MCC indexes are used to evaluate the segmentation accuracy of the forest fire image. In the case of excessive segmentation and insufficient segmentation, the average accuracy of the MD-BPNN algorithm evaluated by using the HAF index is 80.37% and the segmentation result still has good anti-interference performance, thus enhancing the applicability of the algorithm in a variety of scenarios. The average accuracy of the MD-BPNN algorithm is 92.73%, which indicates that the algorithm has high accuracy.

The improved deep learning algorithm improves the efficiency of forest fire risk identification. However, there is still room to improve model performance. In future research, we will further optimize the performance of the algorithm and improve the ability of forest fire risk identification and prevention.

## Data availability statement

The raw data supporting the conclusions of this article will be made available by the authors, without undue reservation.

## Author contributions

SZ and WW: conceptualization. SZ, PG, WW, and XZ: methodology and investigation. SZ and PG: software and visualization. SZ, WW, and XZ: validation and resources. SZ, PG, and XZ: formal analysis. XZ and PG: data curation. SZ: writing—original draft preparation. WW and XZ: writing—review and editing and project administration. WW, XZ, and PG: supervision. All authors read and agreed to the published version of the manuscript.
